# Supplementary material for: “If diagnosed early, you will be stressed and die…” drivers for breast cancer screening services uptake among women in Dar es Salaam
Source: PLOS Glob Public Health. 2024 Nov 4;4(11):e0003390. doi: 10.1371/journal.pgph.0003390 (PMC11534240; doi:10.1371/journal.pgph.0003390)
Supplement: S1 Data — (ZIP) [file pgph.0003390.s001.zip › TRANSCRIPT DATA EDITED/FGD 1-GROUP 1-OLDER WOMEN. Translated.docx]

**FGD-GROUP 1-OLDER WOMEN.**

**TIME: 59:52 MINUTES**

**Interviewer:** So let's start now, shall we? The first thing we want to discuss is the information we receive in our community about encouraging people to come for cancer screenings. I would like to know what sources of information you have in your area or where you typically get this information from. For instance, if ------is conducting screenings for breast cancer, cervical cancer, or any type of cancer, is everyone free to go there and receive these services if they are available? What specific sources of information provided you with this knowledge? Since you all here have already undergone screening, where did you get that information from? Please, number 4.

**Interviewee 4:** (Coughs) The first source is TV; there are often many announcements and advice on how to check yourself if you feel… You are taught how to examine yourself if you find something unusual, like a lump you don’t understand. You are advised to go see a doctor. You can visit a general doctor, but later they might refer you… The second source is that you can go to a major hospital where you can have a more thorough examination and get more certainty. So, we start with TV, I understand that, and then I personally know I need to do that. After doing so, if I discover symptoms, I take steps to see a doctor, and then the doctor encourages me to go to a major hospital.

**Interviewer:** What specific information did you see on TV? What exactly were they talking about?

**Interviewee 4:** They were talking about cancer (interviewer: mm!) and how it is now and what steps to take when you suspect you have a problem like that… how to identify a lump.

**Interviewer:** Please, number 6.

**Interviewee 6:** Thank you. My first source is TV. Later, I had a problem where I was itching in private areas (interviewer: mm!) and when I went to the hospital, every hospital when I went to get medication, I saw it was the same as before (interviewer: mm). In the end, I decided to first go to government hospitals (interviewer: mm!). When I went, if they told me to buy a certain medicine, I would say I had already used it; for this medicine, I had already used it. They said, “Go to a major hospital like Regency, ………………, or Aga Khan.” I went to ……………………i, and when I arrived, I met a doctor who examined me, prescribed some medicine, which I used and felt better. But then the disease came back, and I started itching again. I went back, and I didn't find that doctor but saw another one. The new doctor told me, “Don’t be too brave; you might have cancer.” “You might have cancer,” he said. Since he told me that, I haven’t returned until today. If it’s cancer, I need to go get checked. When I came here (interviewer: mm!), I arrived at the examination room (interviewer: mm!) and told them I had come with a problem—what should I do? They told me, “Come back tomorrow morning to get medicine.” So, when I came back, I started testing for cervical and breast cancer, and it was found that I didn’t have either. The itching I was experiencing was now treated, and I began receiving treatment (interviewer: mm!). You see? The itching was so severe it felt like it was coming from inside, and I also started feeling pain in my knees. I had trouble bending and my vision began to weaken. I noticed my nails weren’t white anymore; they started to fade, and my legs became hard and dry. I saw cracks even on my toes (interviewer: mm!). When I got the medicine and used it, I saw the problems starting to decrease. Once the issues started to decrease, I realized these were the right medicines. So I continued using the medicine and I am thankful that now I am well, and I thank God (interviewer: okay, thank you very much).

**Interviewer:** Mh! How did you find the services here at ------when you came for treatment?

**Interviewee 6:** The services are very good. Whenever someone tells me they are unwell and I listen to them, I directly advise them to come here. I tell them to go to ------(interviewer: mm!).

**Interviewer:** Mh! Number 1, where did you get the message?

**Interviewee 1**: I got the message from the radio (someone coughs). I was at a hospital where they saw there were some rashes in my private areas (interviewer: mm!). When I had an x-ray, they gave me a paper and told me, “Mama, I don’t understand these rashes (interviewer: mm!), I advise you to go to ……...” I came here that morning, got a number, was examined, and it turned out I had no issue (interviewer: mm!). I started treatment and the rashes went away.

**Interviewer:** Number 3, have you ever come for an examination? (nodded head to respond) Number 2, please.

**Interviewee 3:** No.

**Interviewer:** Number 2.

**Interviewee 2:** Number 2, I have previously been treated for a lump.

**Interviewer:** Where did you get the information that you needed to come to ------?

**Interviewee 2:** I heard the information when I went to a health center in ……… because I had a lump that was producing discharge. The doctor looked at me and said, “Go to ------,” so I came here. When I arrived, I didn’t have enough money for the tests, so I had to go back home. I had to return to …………., where they did an ultrasound and said they couldn’t find any problem, and they gave me medication. I went back home.

**Interviewer:** Thank you. Mh! For those of you who have seen TV messages, how do you find them? Do they provide enough information to make someone decide to go to a hospital for screening services? Number 1.

**Interviewee 1:** They are not sufficient because some people don’t have TVs or radios, and others live in rural areas. To raise awareness, maybe using vehicles to distribute flyers (interviewer: mm!) could help. When I first came here, there was a flyer distributed by a passing vehicle (interviewer: okay), and that’s how I knew about it and decided to go directly.

**Interviewer**: Number 6. Ah! Let’s start with number 5. Number 5.

**Interviewee 5:** I didn’t have any information before coming here. But I was motivated by the TV advertisements (interviewer: yes). They said that cervical and breast cancer are deadly for women, and if you’re a woman, you should go to the hospital for a check-up. In our area, the local hospital was announcing (interviewer: mm!) on flyers, saying “All women need to check their cervical health.” They told me to come every Saturday to our local hospital for screening. I went, and they referred me to Temeke Referral Hospital (interviewer: mm!). At Temeke, they said they had concerns, and I needed to get more comprehensive tests. They referred me here for a check-up, which I did in 2018. I paid 18,000 shillings for the check-up, and after a week, the doctor said the results were good but advised me to come for regular check-ups. Due to my limited resources, I didn’t have the means to follow up. The person who helped me was my elderly relative, who passed away in 2019. I had no support and had to ask my children and husband to contribute during Ramadan, but they said, “You brought this on yourself; you went for screening and had no problem, so you should know better.” I managed to gather some funds and came again during Ramadan for a check-up. Thankfully, I had no issues (interviewer: okay).

**Interviewer:** How do you find the services you received? Can you describe them?

**Interviewee 5:** The services are truly good, and we are grateful.

**Interviewer:** What about the language of the service providers, confidentiality, privacy, and their environment?

**Interviewee 5:** The services here are really good. There is confidentiality and a good environment, and we thank God (interviewer: okay).

**Interviewer:** Thank you. Mh! Number 6, did you want to add something?

**Interviewee 6:** Number 6. The advertising services are there, but in rural areas, they are absent. Have you heard? (interviewer: yes). In rural areas, there is a lack of information. A neighbor from Tanga came here with an illness. During our conversation, you might think they have a leg issue, but there might be an underlying problem they haven’t mentioned. You know, women’s private areas are a sensitive subject; women are often shy (interviewer: mm!). They feel embarrassed. They might hesitate to go to a doctor, but they don’t feel embarrassed to see a male doctor (interviewer: even if it’s for breast issues?).

Ah! The real issue is they have a problem down there, but they feel too shy to tell a doctor about their problems. This shyness causes them to delay until they start seeing symptoms like discharge and then realize they have a serious problem. Some people hide their issues and are afraid to speak up. If there were advertisements in rural areas or if there were vehicles and flyers, people would be more aware. Even if some might not read the flyers, hearing announcements on vehicles could make them realize they have a problem and seek solutions.

**Interviewer:** So, you’re telling me that TVs are accessible, but when we come to the clinic, we see that many people don’t come, even those who are not from the villages but from Dar es Salaam itself don’t come.

**Interviewee 6:** Some people, when you tell them to go, will say, “Oh, they are doing this… oh, I don’t want to…” like a duck’s mouth, “I don’t want to,” everyone becomes afraid.

**Interviewee 5:** Sometimes men are an obstacle; as a woman, you have to be strong. (Interviewer - What do men say?) They become anxious; they ask the doctor, “Who examined you? A female doctor or a male doctor?” They question if a man examined you. For example, when my husband and I used to go, I would tell him to stay aside and see if I was examined by a female or male doctor! So, you need to be strong yourself.

**Interviewer:** Hmm. Number 4, do you want to add anything?

Interviewee 4: It’s not that the TV advertisement is sufficient! Yes, as we say, it might seem sufficient, but it is not (Interviewer - Hmm!). Because even here in the city, some people don’t have TVs, and others are very busy; they wake up at 10 pm to cook and return home at 4 am, exhausted (Interviewer - Hmm!). They have to wait until the morning to go back to work. So, they miss the TV time. When we return to the villages, there is no TV, so those who don’t have TVs miss such announcements often. The important thing is that if we were doing this in hospitals, here in regular hospitals, dispensaries, with mothers going to clinics, and those getting treatment, there should be a short period in every hospital and dispensary dedicated to educating people about one, two, three things, such as symptoms and signs. This would encourage people to go to hospitals. Not only in the villages but even here in the city, people go to hospitals just to get treated and leave. But if there were awareness programs, maybe mothers would come to the hospital with one or two symptoms… if there was a special program emphasizing that now we are entering a period of educating patients, whether women or men (Interviewer - Hmm!). Yes, and if you educate men well, they will recognize symptoms and advise their wives to go to the hospital quickly. Some men are reluctant and need assurance; if they understand the symptoms, they will encourage their wives to seek care. For example, when I first told my sister that I felt a lump inside, she advised me to go for a cervical exam. I went to …………, and they said they didn’t have the equipment, so I came here and was tested. I thank God my cervical exam was fine, but the lump became a challenge (Interviewee 4 mentioned his name). They told me to go back and get 50,000 (Interviewer - Hmm!), I went and got it, but later they said the test price had increased to 80,000. I paid 80,000 and was referred to ……………., where the test cost 48,000. After that, they said it wasn’t enough; they needed another test for tissue removal. So, I came back to calculate costs. You wouldn’t believe how expensive it was. If I didn’t have relatives, I would have been in trouble because they said, “You’ll stay in the ward until the money is found.” I didn’t know where I would get the money. So, I was confined to a doctor’s room to start calling relatives, and I’m thankful to the doctors. I’m grateful that instead of removing tissue, they found only fat. So, it could be fat! I thank them for their support in removing the lump. I left the hospital feeling well but was still worried about the costs (someone coughs). I was told the costs, and I felt confused. At ------, I had never received such a painful comment during testing, but at ……….., a nurse asked, “Do you think this service is free?” I said no, and I was prepared for a standard cost, but it turned out to be more expensive due to the need for surgery, which increased my fear of affording it! “Please stay and consider what to do.” I then started calling relatives, friends, and received money. I was directed to the accounting office and kept being sent around, finally returning to pay and collect a receipt. I told my sister that we need improved services because I had to move around alone, relying on the money sent to me. Why not have agents here so that the patient can pay and get a receipt immediately? I could have fallen outside, and you would face another problem. One person said, “Okay, we understand, and we will work on it.” These are the challenges we face. But honestly, education is crucial; providing the knowledge to women is very important. (Interviewer - Thank you)

**Interviewer:** And anyone else with additional comments? We were discussing whether the information is sufficient.

**Interviewee 1:** I already spoke (Interviewer - Did you speak? Okay) that even those in the villages should get it.

Interviewer: Number 2, did you speak?

**Interviewee 2:** I already spoke (Interviewer - Okay) that announcements should be everywhere.

**Interviewer:** Hmm! I would like to ask if you still remember, when you came here to ------for the examination, what information did you receive before the actual examination? Because when you arrive, you meet the service provider who should perhaps give you some information. What information did you receive in the examination room? Was there anything you were told? Hmm! Number 6.

**Interviewee 6:** First, I was told to come in the morning to get a number. After getting the number, I had to wait (Interviewer - Hmm!). Later, I started the briefing and was given instructions, directed on what to do, and informed about everything. Then we were called one by one and told, “Go and register to start treatment.”

**Interviewer:** Hmm! Number 5?

**Interviewee 5:** As my colleague mentioned, when you arrive, you first receive a number and enter a classroom where you are given a seminar on the symptoms of cancer: stomach cancer, breast cancer, cervical cancer, and TV was shown to us for about half an hour. We were then told to proceed with the tests.

**Interviewer:** Yes, number 2?

**Interviewee 2:** As my colleagues said (Interviewer - Like what Number 5 and Number 6 said) Hmm!

**Interviewer:** Number 1?

**Interviewee 1:** I was very pleased with their teaching because they put up posters, on TV, and taught us about which foods to eat and exercise. After that, we could ask questions and they also asked questions. It was a great comfort; you are taught and understand everything there. So, when you leave, you become a good teacher for others (Interviewer - Alright).

**Interviewer:** What do you think should be added after the education you received? Perhaps what else would you like to see added regarding the education? Should something be added, like a leaflet that someone can take away, or what do you think could be added to the class you attended?

**Interviewee 1:** The class we had was sufficient. Maybe for those who were outside, you could give them leaflets so they can read on their own.

**Interviewee 5:** I remember that years ago, when we came in 2018, they used to give out leaflets, and I would take them home to read. At that time, they hadn't set up any TVs yet.

**Interviewee 1:** But I’m grateful because, despite using the leaflets, I went on to encourage my relatives, and almost all of them came for cervical screening and started treatment for liver protection.

**Interviewer:** When you first educated your relatives, what was their reaction?

**Interviewee 1:** They did not understand. They did not know if such a condition existed or if there were dangers like those.

**Interviewer:** But after you explained to them?

**Interviewee 1:** After I explained, they said, “Are you sure?” I told them I was sure and explained that if you get it, you cannot recover, but if you have protection, it means it can be treated even if you get it.

**Interviewer:** So, does it mean that we ourselves can be sources of information for others?

**All:** Yes, very much. They can be good teachers.

**Interviewer:** Moving to Number 4.

**Interviewee 4:** I was referring to the issue of information sharing. For example, on the first day, when I was told to go to ------, I told my sister that I was heading to ------. She was shocked and said, “What are you going to do there?” I explained that at ------ they said there were no facilities for that service, so I would go there. I was reassured. “When you return, let me know.” I came here, and as you said, we were educated by the nurses on a few things. After that, you take a number and wait for your turn. I thank God, you are tested, and they tell you, “You are doing well, we are glad you are improving.” They really treat you well and educate you thoroughly. So, the challenge, as my colleague mentioned, is that if you want to educate your relatives, you must start telling them. I went there, started being tested, but it is something normal because once diagnosed, it is easier to get proper treatment. However, the issue of going to ------seems daunting to many.

I am grateful because I have already come, even though there are aspects I am not used to and still fear. For instance, when I arrived in the morning, I saw the X-ray machines, and seeing how people talk about X-rays in the streets, it adds to my fear. So, education is necessary! There are things that go on… For example, there was a time I wanted to have a specific type of education. You wouldn’t believe, there was a relative of mine, a child, whom I called asking for health advice. They said, “We are concerned; we need to do more tests.” You wonder if you are ready to be told you have cancer or not? They were in class that day and couldn’t respond immediately. But I wished I had someone to educate me, to alleviate my fears. I really wanted someone to tell me, at least a doctor at Muhimbili, “When you are told this and that, it means this,” but I missed that guidance to relieve my fears.

**Interviewer:** So, you want to say that when providing this education, we should make it clear that if additional tests are needed, what does it mean? So that people are not afraid? (All - Yes, they should be prepared.)

**Interviewee 2:** When you come to ------, people think it’s the final stage. “Oh! You’re going to ------.”

**Interviewer**: “Going to ------” is how people perceive it?

**Interviewee 5:** For example, when I came from the district referral hospital and was told I needed to go to ------, I thought, “This must be a serious illness.” When I received the results, I came with my sibling, and I told the doctor I was told I had no problem. We were surprised and embraced each other. Even during Ramadan recently, my sibling accompanied me, showing that “------” has a reputation of being threatening.

**Interviewee 6:** It’s true that many people fear that name. Just mentioning ------makes people think you already have a serious condition. They know that once you are diagnosed with cancer, you go there; they assume you already have it!

**Interviewee 4:** Just mentioning the name alone… Even when I received your call while I was in Morogoro, I told them my doctor is at ------. They asked, “What’s going on?” They know that there is a serious issue.

**Interviewer:** Wow!

**Interviewee 5:** Even when I told my husband I received a call from ------, he asked, “You said there was no problem; what’s going on?” I said there are other research issues we need to address, not just for me but for others as well. It’s truly a challenge.

I**nterviewer**: Moving to Number 1.

**Interviewee 1:** But people in the community mislead each other a lot. You’ll hear, “So-and-so was told they have cancer.” They say, “Don’t go for radiation; it’s fatal. Use traditional medicine.” There is a lot of misinformation.

**Interviewer:** Explain clearly about traditional medicine. Does a person decide to use it after they have been tested or before going for the examination?

**Interviewee 1:** After being tested and found to have cancer, for instance, cervical cancer, or breast cancer, or any other type, you hear, “Let me show you traditional doctors; you’ll use roots and be cured.”

**Interviewer:** So, the fear is not only about going for tests but also continues during treatment?

**Interviewee 1:** Yes! The fear during treatment is even greater. The act of leaving home to come here for testing is a significant challenge. It’s a very big test (Interviewer - Okay)

**Interviewer:** What do you think religious and government leaders can contribute specifically in delivering these messages and information about diseases? Hmm!

**Respondent 4:** Thank you. You reminded me, because I had raised my hand to discuss this but ended up adding so much that I forgot. The role of religious leaders in mosques and churches, even those who meet in open spaces, is very important. If they were given the chance to talk about these issues, it would be very beneficial. For instance, in church, if there were times set aside to educate the community with messages like “We are advised to go for testing, let’s go and test for things like HIV, cancer, and tuberculosis,” it would be helpful. Leaders would be serving both spiritually and physically by giving such advice. So, religious leaders’ encouragement would help raise awareness.

**Interviewer:** Number 1, you raised your hand.

**Respondent 1:** I wanted to say the same thing, that religious leaders are very commendable. They teach both spiritually and physically and are highly respected by their followers.

**Interviewer:** Is there any way they might mislead?

**Respondent 1:** There’s no way that a leader would mislead.

**Interviewer:** If we talk about a well-known religious leader who claims that people come to him for healing, how do we address that?

**Respondent 1:** Ah! If a religious leader is known to claim they can heal everything, they might tell people to go for tests first and then come to them. However, they might say they can heal after diagnosis.

**Interviewer:** Ah, so treatment would be sought there?

**Respondent 1:** I heard one person’s testimony about throat cancer. They went for a test, were told it was completely damaged, went to be prayed for, and were healed. That’s their faith.

**Interviewer:** How do you think society perceives it when someone goes there with cancer and gets healed? How do you view it personally?

**Respondent 4:** Due to fear of illness and death, if I’m told I have cancer and then a pastor tells me, “I’ll pray for you, you might get healed,” I wouldn’t miss going. It prepares me to take the decision to go, but while going, I’m aware that I’m seeking traditional medical treatment. If I don’t understand, I would need the doctor to clarify if I will actually be healed or if I’m being deceived.

**Interviewer:** Number 1?

**Respondent 1:** If I’m told by my leader that I’ll be healed if I have faith because God is there, I believe in healing through prayer, but I will continue with my medical treatment and prayer.

**Interviewer:** So, what specific contributions can our religious and government leaders make to encourage people to seek services at health centers or not? What positive and negative contributions can they make? We’ve discussed how they can encourage effectively.

**Respondent 4:** For example, as we discussed the well-known religious leader who says they can heal through prayer, on the other hand, a priest might advise you to go to the hospital if you’re already diagnosed with cancer. There are different perspectives.

**Interviewer:** Let’s talk about community perceptions. How does the community view someone who goes to a hospital for breast cancer and cervical cancer screening? For example, if you told your mother or sister and they were very scared, how does the community view it? How do they respond to the message you bring? Number 2.

**Respondent 2:** When I take the message home, they perceive it as fear. They believe that if they test and find out they are sick, they will not recover and will die.

**Interviewer:** Number 6.

**Respondent 6:** I don’t have much to say, but some people avoid testing until the problem becomes serious. At first, they hide it until it becomes visible. Many people think cancer is only a women’s issue, and men believe they don’t get cancer, but they too need to be encouraged to get tested.

**Interviewer:** Any opinions on how to improve the dissemination of information and education to ensure more people receive this information effectively? If anyone has additional opinions before we move on, please share.

**Respondent:** No more comments.

**Interviewer:** Let’s discuss the cancer screening services themselves. What is your opinion about these services, whether for cervical or breast cancer? Do you think this service is beneficial?

***Respondent 6:*** Yes, it is beneficial. It helps you know whether your health is okay or if you have cancer. If you delay and remain silent, even minor problems can become severe and harder to treat.

**Respondent 5:** Or you might stay silent due to fear, thinking any discomfort is the same thing, but when you get tested, you might find out you’re safe and continue with other aspects of your life.

**Interviewer:** Based on your experiences, Number 6 and Number 5, how did you find the service when you used it?

**Respondent 6:** The service is good, and the doctors are also good. They provide good advice and information. They don’t have negative comments, and we are thankful for that.

**Interviewer:** Number 4, what is your opinion?

**Respondent 4:** My opinion is that it is good to get tested to know if you have a problem or not. The challenge arises when there is a lack of understanding. If you’re told you have symptoms, it’s better to be told you’re fine than to have symptoms. The education is not always complete.

**Interviewer:** How about your family’s perception when you tell them you’ve had this service?

**Respondent 4:** Initially, everyone was fearful, but later, when I was told the lump was just fat, they calmed down.

**Interviewer:** How does your family view coming for this service?

**Respondent 4:** They see it as educational, but it often takes some time to overcome the challenges. The more you live with people and share your experience, they become more understanding. When they see you have been tested and found safe, they are more likely to take action if needed.

**Interviewer:** Number 3, what’s your opinion? Have you had any experience with breast cancer screening?

**Respondent 3:** I’ve learned from it, and now if I notice symptoms later, I won’t be as scared.

**Interviewer:** Number 2, what do your close family members think, especially if you told them you had a breast screening?

**Respondent 2:** I went for a test at ----, which was expensive. They checked and said there was no problem (Interviewer: so you went to ------but were told there was no issue at ……..?). (Respondent 2 is not audible)

**Interviewer:** Number 1, what’s your opinion about your own and your family’s views?

**Respondent 1:** My opinion is that I have advised my friends, and they have received the vaccine (Interviewer: about screening?). I want to convince my husband to get tested as well. (Interviewer: So at home, they are receptive and don’t have negative views?). They don’t.

**Interviewer:** Let’s talk about the general acceptance of this service. What factors influence people to get tested or not? Number 1.

**Respondent 1:** Factors that encourage people include telling them about the benefits of early testing versus delaying. It motivates them to act quickly. (Interviewer: What discourages them?) Many times, it’s a lack of understanding. If they had someone to explain things properly, even if they don’t have money, they would still find a way to pay for the tests.

Interviewer: Number 4, what about challenges that could either encourage or discourage someone from coming for screening?

**Respondent 4**: Educating someone helps them understand that early detection allows for easier treatment, while late detection is more severe. Challenges might include being told to come back another time or not having enough money to pay for the tests.

**Interviewer:** Number 6, what encourages or discourages people from coming for cancer screening?

**Respondent 6:** Sometimes, the way you inform them and the reception they get matters. For those under 50, tests are free. Informing them clearly about the benefits can encourage them. However, lack of money remains a significant barrier.

**Interviewer:** Hmm! Is there anyone else who would like to contribute? Perhaps we could discuss the environment here at our screening facility. Looking at how it is set up, does it seem intimidating to you? Please, go ahead.

**Interviewee 4:** You’ve really touched on a point. Because initially, before I came to the hospital, listening to stories about this hospital from my country, I was told that it’s a place to be feared. I was anxious about what I would find. People would say, “------isn’t good; if you go there, you’ll see people with wounds, people with serious conditions.” The stories made it sound so scary, like ------was a hospital only for severe cases. But when I arrived here, first, I walked around and then was directed to room 11, but I ended up in room 10. When I went inside, it was not as I had imagined. Even the x-ray room was not as intimidating as I had expected. It was clean and well-organized. You have air conditioning, and you’re in a clean environment where you wouldn't even know who has a problem. I was pleasantly surprised by the warm reception and the quality of service, similar to other hospitals.

**Interviewer**: How about the time spent on service? From the moment you arrived until you were attended to, how long did it take?

**Interviewee 4:** The service time depends. If you are the first, you will be served first. If you are the fourth, you will be served fourth. They don’t have the habit of prioritizing someone over another without reason. Number one goes in first, and if there’s another person with the same number, they will let them go ahead before you. The procedure is quite orderly.

**Interviewer:** What about distance? Is the location of the center a barrier for people?

**Interviewee 4:** Yes, that is a challenge. For example, I come from …….., specifically from ------------. Traveling to and from here costs about 6,500 TZS. When I was undergoing tests, the cost was significant, but I am grateful I managed. It involves taking a bajaji from ……i to here, which is quite a journey. I told my husband that I needed to return to ------, and he said, “You know it costs money.” I said, “I know, but I need to go.” We women often add a little extra to our savings for such expenses. I had to explain to him that I needed to go to the hospital because I might get some important information. Even at home, money is a challenge, and getting from Mbezi to ------is a significant cost.

**Interviewer**: What do you think should be done about this?

**Interviewee 4:** For example, we have hospitals in ……… and…………., and there is another one nearby at ------. From where I stay, I can see----------. Even though it’s a bit of a detour to reach ------, it’s a longer journey, but closer than ------. If more local facilities could provide the services offered here at ------, it would help a lot.

**Interviewer:** Please, Number 5, what are your thoughts on these challenges and how we can improve access to services?

**Interviewee 5:** I think we should focus on providing initial services, such as cancer screening, and make sure these services are available regularly each year. This would be very helpful.

**Interviewer:** Is there anyone else with different opinions or additional points we haven't covered? Please feel free to share. Number 1, go ahead.

**Interviewee 1:** For example, hospitals like ------ do provide preliminary screenings. They might suggest treatments if they see signs of cancer. If the situation is severe, they refer you to ------. However, if it’s not severe, they might handle the treatment there. I appreciate that even though many people go to the hospital, the services are good and efficient.

**Interviewee 1:** If you want to understand how serious cancer is, go to the wards.

**Interviewer:** Thank you all for your participation. I assure you that the opinions you’ve provided will be kept confidential but will be used to inform those planning these services. Your feedback will be used to improve service delivery as you have suggested in this discussion. Thank you very much. (All interviewees: Thank you very much.)
