# Supplementary material for: “If diagnosed early, you will be stressed and die…” drivers for breast cancer screening services uptake among women in Dar es Salaam
Source: PLOS Glob Public Health. 2024 Nov 4;4(11):e0003390. doi: 10.1371/journal.pgph.0003390 (PMC11534240; doi:10.1371/journal.pgph.0003390)
Supplement: S1 Data — (ZIP) [file pgph.0003390.s001.zip › TRANSCRIPT DATA EDITED/FGD 2-OLDER WOMEN.docx]

**FGD 2 GOUP 2**

**TIME: 39:6 MINUTES**

**TRANSCRIBER: ……………………**

**Interviewer:** First of all, thank you very much for taking the time to come and listen to us. I would like to start by understanding what you know about early cancer screening services before symptoms appear. What is your understanding of early cancer screening, and how is it perceived in your community? Number 5, please go ahead.

**Interviewee 5:** Thank you very much. For example, in ------, there is a community organization called…(---- Outreach Program). They hold special campaigns every year to inform people that cancer awareness day is on October 6th. They announce this and encourage people to come for screening. I remember participating in one of these campaigns in 2021. I went for a check-up and found no signs of cancer. However, later on, I discovered a lump and decided to seek medical help myself.

**Interviewer:** Ah! I see. Let's move to another participant, Number 3.

**Interviewee 3:** Yes, the doctors at the hospital advised me to self-examine. They said that if you feel any unusual lumps while lying down, you should go to the hospital for further examination. I followed this advice, and although I initially felt a small lump, I ignored it. Later, my husband, who was handling hospital matters, suggested I see a doctor. When I went to …….. hospital, they found the lump and decided to send it to ------ for further examination. The results indicated that it was not cancerous, but I was advised to start treatment at ------. After several treatments and surgeries, I am now attending regular check-ups.

**Interviewer:** Thank you. Number 2?

**Interviewee 2:** Honestly, I never thought about cancer problems before. I only heard about it but didn't know it could affect me.

**Interviewer:** Have you ever heard about early cancer screening?

**Interviewee 2:** Yes, I have heard about it. I first learned about it in 2021, and I noticed some symptoms on my breast.

**Interviewer:** Let's continue. I would like to know your understanding of early cancer screening services before symptoms appear. What do you know about early breast cancer screening before symptoms start?

**Interviewee 2:** Honestly, I only heard about it. I didn’t understand much about cancer symptoms.

**Interviewer:** Number 1?

**Interviewee 1:** Before I became ill, I had no knowledge of cancer. I had only heard about blood cancer within my family, so I only knew about that. I had no understanding of breast cancer specifically.

**Interviewer:** Thank you. Returning to Number 5, you mentioned understanding early screening. What benefits do you think early screening provides? Can you explain the advantages you have seen and who should be screened?

**Interviewee 5:** Early screening helps in identifying potential issues and can benefit the community. For example, when I returned to ------ for a check-up and found a small lump, they advised us to spread the message through local leaders. This resulted in many people coming forward for screening, including those who might not have been aware of their condition.

**Interviewer:** What did they say about who should be screened?

**Interviewee 5:** Anyone can be screened for breast cancer, regardless of age or gender.

**Interviewer:** Thank you. Number 1, who do you think should specifically be screened for early cancer detection before symptoms appear?

**Interviewee 1:** Women who have started menstruating should be screened regularly. They should examine themselves and ensure that there are no lumps before menstruation begins.

**Interviewer:** How do you currently carry out self-examination? Given that you have experienced illness on one side, what efforts do you make to ensure the other side is safe? Number 5?

**Interviewee 5:** We closely follow the doctor’s advice. It is important to listen to the advice and sometimes follow dietary recommendations to prevent the spread of cancer cells. Regular self-examination helps in monitoring any changes.

**Interviewer:** Number 1?

**Interviewee 1:** If you have started menstruation, you should regularly examine your breast for any lumps. If you feel any lumps, consult a doctor.

**Interviewer:** Number 2?

**Interviewee 2:** Like Number 1 mentioned, doctors often advise us to examine ourselves. If you notice any symptoms or changes, you should promptly see a doctor to discuss them.

**Interviewer:** Thank you! Moving on, how accessible are early screening services in our community? Can you discuss how available early cancer screening services are before symptoms appear?

**Interviewee 5:** First, there are vaccination campaigns, which I believe are for children under 14 years old. I am not entirely sure about the safety of these vaccines, but I trust the doctors. Additionally, organizations like ------ have been working hard to ensure people are screened. They use various methods to promote screening and have been successful in increasing participation.

**Interviewer:** How accessible are these early screening services in your community currently?

**Interviewee 3:** For example, in our area, after a screening, you are advised to continue self-examinations at home.

**Interviewer:** Number 1?

**Interviewee 1:** In ….., there is a lot of awareness. The government and media are doing a good job. During the month of May, there are frequent broadcasts about cancer awareness, which helps in spreading the message.

**Interviewer:** Number 2, how are the services there?

**Interviewee 2:** The government has been proactive. For the past two months, they have been going door-to-door to encourage people to get screened for breast cancer and cervical cancer. Many people have participated, and even I went for screening to check my cervical health.

**Interviewer:** Number 5.

**Respondent 5:** In the past, there were organizations like ….., which is a women doctors' association. They were very active in campaigning about cervical and breast cancer.

**Interviewer:** Thank you very much. Let's move on to another question in this area of initial screening. Are there any challenges that your family members, friends, or neighbors face that prevent them from going for breast cancer screening or initial check-ups?

**Respondent 1:** It's fear, you see? Many people are afraid. When they see someone is sick, they think, “Oh! I’m not going to seek help.” They are filled with fear and say, “I can't go.” They view the term cancer as something very severe. They think cancer is untreatable, but it’s like any other disease. You could get malaria and die, or you could have cancer and survive, but understanding is limited and how they perceive it.

**Interviewer:** Hmm! Number 5, do you have anything to add?

**Respondent 5:** I don’t have anything to add.

**Interviewer:** Hmm!

**Respondent 2:** Honestly, in my family, there are people who suffer from painful breasts, but whenever I advise them to go to the hospital for a check-up, they are very fearful. They tell me, “Even if,” meaning they don’t fully accept the advice. They might say, “You could go there, get checked for free, and if you find any issues, you’ll get treatment early. It’s better than sitting with fear and delaying, which could lead to a bigger problem.” So they are there, but every time I advise them, no one understands, they are there.

**Interviewer:** So, what is the major issue?

**Respondent 2:** The major issue is fear! Fear is a big problem. When people see me, they see I had a surgery, so they are afraid to go to the hospital. They don’t want to hear about it. I try hard, but I fail.

**Interviewer:** Okay! Perhaps Number 4, do you have anything to add?

**Respondent 4:** In my family, speaking from my experience, my close relatives—like those in my household—face challenges. As I mentioned earlier, when I was diagnosed, I went to the hospital, and they told me to go to ------. They said, “------, call them. They will tell you that cancer is just like any other disease. Don’t worry, you will be treated, you will recover, and life will go on.” So, I went to ------ for tests, but the results took a long time. My relative said, “It’s better to get treated at a private facility where we’ll spend a week than wait seven months.” So I was treated on……... I had the surgery and was out in just three days. Honestly, the surgery was not painful; it was the chemotherapy that was hard. After chemotherapy, my condition improved, and I continued with my daily activities until I had a recurrence. But I haven’t been abandoned; my family supports me through it all. People do get treated and recover. Even if someone dies from malaria, does that mean cancer is a death sentence? You will be treated, you will recover, and life will go on.

**Interviewer:** Have they ever come for initial screening to know their condition earlier? How do they view this?

**Respondent 4:** The challenge is fear. Even if you tell someone to check for lumps, they might respond, “I’m afraid of the pressure of finding something.” I try to encourage them, but it remains very difficult for them to understand. Fear is the main issue.

**Interviewer:** Okay! Thank you. Now, based on your opinions, what do you think should be done to help the community so that more people can go for early health checks, especially for breast cancer, which is on the rise? What methods should be used?

**Respondent 4:** I think more engagement with community leaders and organizing events regularly is essential. If people see that their leaders are involved and see regular events promoting awareness, they might become more receptive.

**Interviewer:** Moving on to Number 3.

**Respondent 5:** Thank you. I believe that if the government took the same vigorous steps it took for HIV/AIDS for cancer awareness, it would be beneficial. The HIV trend may be decreasing, but the cancer trend is still high. We all didn’t expect to encounter cancer, but now some people are at home fearful, and others don’t even understand what cancer is. The government should put in the same effort for cancer as it did for HIV/AIDS by using media to educate people on what cancer is. When people understand, their fears will diminish.

**Interviewer:** Number 1.

**Respondent 1:** Even in companies and workplaces, doctors should visit and inform employees. Since these workers are in contact with the public, the information will reach families and communities. Every gathering should include discussions about breast cancer challenges.

**Interviewer:** Thank you very much. Number 2.

**Respondent 2:** Even if they do what others are doing by going door-to-door, community leaders should spread the message. Many people will be motivated and will go to hospitals to get checked. By continually talking to people every day, they will gradually lose their fear.

**Interviewer:** Thank you very much. I would also like to know where you receive information or messages about breast cancer screening. Where did you first hear about these services?

**Respondent 5:** I have heard about it on TV and radio. In the past, there were campaigns where they came to ------ to check for cancer. I heard about it before, but as I mentioned, people were involved in it.

**Interviewer:** Number 1.

**Respondent 1:** Through radio programs, especially during Women’s Day and cancer awareness programs, you get information.

**Interviewer:** Hmm! Okay! Number 2?

**Respondent 2:** Initially, I heard about it when they came to our area for screening, but I didn’t know what cancer was. I didn’t hesitate; I went for screening even though I didn’t fully understand it. That was around 2015. I went with my sister and didn’t fully grasp what was being done, but I went because it seemed important. They checked for lumps, and the results were fine, but I didn’t return until I encountered a problem later.

**Interviewer:** Are these messages sufficient to make someone understand or motivate them to seek services? Number 5.

**Respondent 5:** The messages are sufficient; they are well-organized and communicated. ------ also makes efforts by providing many announcements and campaigns.

**Interviewer:** Regarding the announcements, do they provide enough information for someone to get all the details?

**Respondent 1:** ------ ensures that people who have had cancer and are now recovering share their experiences, explaining what cancer is and what actions to take. It’s up to the individual to accept or reject the information.

**Interviewer:** We were discussing whether the messages are enough to encourage people to come for screening. Alongside that, how do family, close friends, and the community view this information? Do they see it as relevant or how do they perceive it?

**Respondent 5:** I’m not sure how to put it, but some families understand what cancer is and know what to do. For example, my husband is very health-conscious, and when I wanted to go for a test, he said, “Go to ------ and get your health checked.” So, people do understand. Additionally, when I had a lump, he encouraged me to go to the hospital, so he understands.

**Interviewer:** Number 1.

**Respondent 1:** There are family members who understand and support someone with cancer, offering encouragement and standing by them through tough times.

**Interviewer:** Do they view this as a personal issue that requires them to get checked?

**Respondent 1:** That’s where the challenge lies, as we discussed earlier. The major issue is fear.

**Interviewer:** But do they believe the service is important for them?

**Respondent 5:** Yes, it is important. When people understand the education provided, they will seek treatment earlier and prevent deaths. Just like the campaigns for mothers and children, a special campaign for adults could reduce fear.

**Interviewer:** Number 2. How do close relatives perceive the information about cancer screening campaigns? Do they view it as important or relevant to them?

**Respondent 2:** For families, they acknowledge that the problem exists, but the challenge is still the fear of getting checked. Even if you suggest a check-up, they fear that it might reveal a problem. They would rather stay as they are than face the fear of a diagnosis.

**Interviewer:** Based on your views, what can be done to alleviate this fear? How can we better present the information to reduce fear and encourage people to seek services?

**Respondent 5:** I think cancer institutions should have more psychologists because many people are discouraged by the belief that cancer is incurable. Bringing someone back to a normal state is not easy. If the government or cancer institutions include psychologists, it would help. Even if funds are used for campaigns, people hesitate due to fear. A psychologist could help address these fears effectively.

**Interviewer:** Thank you very much. Number 2, how can we reduce fear?

**Respondent 2:** To alleviate fear among our relatives, neighbors, and friends, we need to continuously encourage them. Even if we keep telling them to get checked, many are still hesitant. We need to keep talking to them daily to reduce their fear and help them understand that getting checked early can lead to better outcomes.

**Interviewer**: Number 4.
**Interviewee 4**: People's perspective will be a bit difficult. They will have to undergo testing (interviewee laughs).
**Interviewer**: When you first heard about getting tested for cancer, what was your initial thought?
**Interviewee 4**: People still have fear; they believe that people with cancer cannot be cured.
**Interviewer**: Finally, I would like to know if you have any recommendations for us, the committee, regarding these discussions?
**Interviewee 5**: I would like to meet with President ----- and suggest that the cost of cancer treatment be reduced. People are unable to continue their treatment due to financial constraints. For example, when you go to the hospital and are told to pay 280,000/=, it's a lot of money. I can afford it, but some people can't. There are many of us here who are not in dire financial situations. I would like to see a day when cancer treatment is free, like with HIV. I compare it to HIV; the costs should be reduced so that more people can receive treatment. Many people are stuck because they don’t have money, and some families have abandoned them. They feel marginalized and receive little support. I wish one day cancer treatment could be free.
**Interviewer**: Thank you. Number 2, how do you view the situation when you tell people to go for testing? What is their perspective?
**Interviewee 2**: Hmm! My family, honestly, I still see a lot of reluctance. For example, just recently my brother's wife called me; she is suffering a lot from a painful breast. She called me for advice, asking, “Sister, what should I do?” I told her, “You should go to the hospital. You should take her yourself; she can’t go alone. You need to accompany her and explain the situation to the doctor.” She said, “Okay,” but I haven't heard back from her since. She mentioned going to ------because the doctors are there. I don’t understand why, as I have heard that this hospital treats cancer. So, they really don’t want to go there, which is why I advised her to go to ------, as I also started there.
**Interviewer**: Is there anything else to add regarding cancer screening and treatment in general?
**Interviewee 1**: Another point to add is that there are many issues related to this. Just like malaria, which is a widespread issue, the government should continue to announce this daily so that people are not afraid to go for breast screenings. We should also continue to encourage those we see.
**Interviewer**: Thank you. Number 4, do you have anything to add?
**Interviewee 4**: No.
**Interviewer**: I think we have finished our questions. I would like to thank you all for your participation and for the valuable insights you’ve provided. If we have more questions, we will contact you. And if you have any questions for us, feel free to call us as well. Thank you very much. Ah-ha!
