# Supplementary material for: “If diagnosed early, you will be stressed and die…” drivers for breast cancer screening services uptake among women in Dar es Salaam
Source: PLOS Glob Public Health. 2024 Nov 4;4(11):e0003390. doi: 10.1371/journal.pgph.0003390 (PMC11534240; doi:10.1371/journal.pgph.0003390)
Supplement: S1 Data — (ZIP) [file pgph.0003390.s001.zip › TRANSCRIPT DATA EDITED/IDI 04 Non screened.rtf]

IDI-Non screened 04
Time 30 Minutes 
Transcriber ………………..

Interviewee: Cancer has also affected our family (eeee). I have a younger sibling who passed away from cancer (mmmhm). He had bowel cancer and was treated at ------(aaa). He underwent surgery three times to remove parts of his bowel (exactly). Mmmhm (aanha), they cut his bowel three times, but that last time was the end because very little bowel remained. He could only eat a couple of spoons after that, and when it returned, it spread a lot. So I wanted to ask, does it spread?
Interviewer: Yes, cancer (mmhm). If you notice a small lump, you might be able to remove it, but you also need to use radiation and possibly chemotherapy because aaa the blood circulates throughout the entire body (eee). So even the cancer cells are carried by the blood and can move to other parts (that's right). If it goes to a place where conditions are right for it to grow, it will start again in a new area. So cancer, even if it started in the head, can reach the cervix, liver, or heart. If you have cancer, even if it started somewhere else, you must receive treatment like chemotherapy or radiation to completely eliminate the cancer cells in your body. Maybe you were just a bit late...
Interviewee: I don't know, because it spread from his abdomen to his leg.
Interviewer: Aah, to the bone.
Interviewee: Eeee.
Interviewer: It happens, especially since blood is produced in the bones. As the blood circulates, it can reach the bones, and that's where the cancer can start growing again. That's why it spreads.
Interviewee: Aaah, okay.
Interviewer: Let's start our discussion (okay). I would like to first understand your knowledge regarding early cancer screening. What do you know about early cancer screening?
Interviewee: Honestly, from listening to announcements, I heard a lot about breast cancer (mmmhm). If you notice any lumps, even if they are small (mmhm), you should examine your breast. If you feel a lump, even if it's far away (mmhm), you need to go to the hospital for a check-up (mmhm). If detected early, it's much better; it can be a great help compared to delaying. You shouldn't underestimate such things (mmmhm).
Interviewer: Where did you hear that announcement?
Interviewee: There was a time when various people went to different places announcing that women could get screened a long time ago (eee). Honestly, I've forgotten the year (mmhm). But many people were unaware (eee), you know, they underestimated the issue. Many went, but a lot didn't take it seriously (mmmhm). They were too busy with their lives; many still don't understand that cancer is a very serious disease, worse than even HIV (mmhm). Cancer is a very serious illness, but many still don't realize that, even if you announce free screenings, people might not take it seriously because they don't feel sick. So I still see that people lack understanding about this disease; many still don't comprehend. Because the disease can progress without you realizing it until you start suffering, then you struggle when it's already severe (mmhm).
Interviewer: Do you think early screening has any importance?
Interviewee: It is very important (mmhm). It is very important because if you catch the disease early, it is easier to get treatment than if it has progressed. Treating it becomes very difficult, and recovery can also be a challenge. You might recover or you might not, depending on how far it has spread or how severely you've been affected (mmhm). Many people go to the hospital when they are already severely affected (mmhm). I've been to ------a lot while my younger sibling was hospitalized (mmhm). He was admitted multiple times; at least three times, and every time he was discharged, he would return after two months. Whenever I left home, I would take him food, so I've seen many patients with various types of cancers (mmhm). But many come when it's too late, which makes their recovery difficult. My younger sibling was in a critical state when he was diagnosed with bowel cancer. One family member was feeling unwell and was taking hospital medications, eventually turning to traditional medicine until his condition worsened, and he was taken for further examinations where they discovered he had bowel cancer (mmhm). I wanted to ask (mmmhm), can cancer be transmitted from one generation to another?

Interviewer: That depends on what type of cancer it is.
Interviewee: Because my grandmother also died of colon cancer.
Interviewer: Well, cancer can also be hereditary.
Interviewee: Really?
Interviewer: Yes, it can be inherited through generations, from grandparents to grandchildren and their children.
Interviewee: I understand that now.
Interviewer: Now, can you discuss the availability of early detection services?
Interviewee: I don't quite understand the question.
Interviewer: I mean, what do you think about the accessibility of early detection services? Where can one go to get checked before symptoms appear?
Interviewee: Honestly, I don't know much about it. Even though we had a patient like that, I still don't know anything. Perhaps if someone notices a difference, they might go directly to Ocean Road, but I don't know if there are centers where one can go for early detection, or how one would access that. Many people are unaware of it.
Interviewer: Have you heard announcements about cervical cancer and breast cancer screenings being offered for free at health centers?
Interviewee: Honestly, I haven't heard that. I can say that I haven't. What I was telling you is that I heard about such announcements a long time ago when they were promoting it in various places. People were told that screenings were free. A long time ago, I even went for a breast cancer screening and was found to have no issues, but since then, I haven't gone back for any other check-ups.
Interviewer: Absolutely. Are there any challenges that people, including yourself, might face in going for screenings?
Interviewee: I think there are no challenges preventing someone from going for screenings. However, many people get caught up in their daily lives, without realizing there are diseases that require early examination before one gets sick. Many don't have that mindset; as long as they feel fine, they simply go about their daily activities without considering the need for checks.
Interviewer: So, the main challenge seems to be education and awareness; people don't know where to access services.
Interviewee: That's true. Let me tell you, for instance, regarding HIV, many people are more attentive. If someone has a slight fever, they start to worry, thinking, "Why does this fever keep coming back?" They become anxious and want to get tested early, as they understand that this disease is serious and can cause fear. Many are ready to test as soon as they feel unwell. Testing has become easier as health workers even visit homes, and testing is available at clinics and hospitals.
Interviewer: Right. Since cancer is a more serious disease than HIV, would you suggest a campaign to raise awareness about early detection for cancer?
Interviewee: Yes, exactly. I hope that such a campaign could be organized to make everyone aware of the importance of checking their health and seeking treatment early if they notice any issues.
Interviewer: But some people take announcements lightly; they hear about screenings but still don't go. What can we do about that?
Interviewee: It's true that many people take it lightly. You might see an advertisement for cancer testing, and they might not show up.
Interviewer: What can we do about that?
Interviewee: Because they don't understand how serious this disease can be. They still don't grasp the extent of the danger.
Interviewer: Thank you. So, you mentioned there were announcements about screening services. What exactly were they promoting, and how effective were those announcements in encouraging people to get tested?
Interviewee: The announcements were primarily for women's breast cancer screenings. They promoted it widely and went to various regions offering free screenings for breast cancer. They were announcing it daily on TV and radio, so even in rural areas, people heard about it. They encouraged women to check their breasts for any lumps and get a thorough examination if they found anything. This information was widely circulated, and it had a significant impact.
Interviewer: Okay. Regarding early detection, do you believe there are real benefits to being screened early?
Interviewee: I definitely see great benefits in that. It's much better to detect this disease early so that treatment can be administered and there's a higher chance of recovery than waiting until someone is severely affected.
Interviewer: How do you perceive your own risk? Do you feel you or your family is at risk for cancer? After caring for a family member who passed away, how does your family view cancer risk now?
Interviewee: Honestly, I must say the truth. Since people don't know the symptoms of cancer or what causes it, it's very difficult for families. We have lost two people to this disease, but even now, we don't understand what kind of cancer it was. We find ourselves asking questions but not getting answers about what type of illness it is or what causes it. This leaves us in confusion, not knowing how to identify or protect ourselves against the disease.
Interviewer: That's understandable. What is the general perception of cancer among people? Do they have beliefs about its causes or any misconceptions?
Interviewee: About 95% of people have misconceptions. They find it hard to recognize the symptoms of cancer, and it's difficult to know if one is suffering from it. Many rush to the hospital only when they have visible issues. For instance, if someone notices a lump on their leg and it grows to a sore, they may think it's something simple and seek general treatment, not realizing it could be cancer. When the situation worsens, they may finally return to the hospital, only to find out it's cancer, and at that point, it may be too late for effective treatment. This lack of awareness leads many to delay seeking help until it's critical.
Interviewer: Thank you very much. If you have any more questions or anything else to discuss, feel free.
Interviewee: My question is whether cancer can affect any part of the human body.
Interviewer: Even the eye.
Interviewee: Yes, even the eye. It can occur in the breast, leg, or even the nose.
Interviewer: ...Even the tongue.
Interviewee: Yes, even the tongue. I wanted to ask, how can you experts tell if someone has cancer? If you can identify it early, how can you be certain that it's cancer before it progresses?
Interviewer: It's not easy. The early symptoms of cancer can be subtle. For instance, someone might notice swollen lymph nodes, or a sore that doesn't heal.
Interviewee: Swollen nodes that may or may not be painful.
Interviewer: They might not hurt but can swell.
Interviewee: Right, just swelling. It can also occur in breast tissue, where a lump might form that doesn't hurt. This could indicate cancer. Sometimes, in other areas, there could be changes like redness or tenderness without swelling, which might also be cancer. It's important to be aware of any changes, especially in the cervix, where early signs can be detected through screenings. Have you ever been screened for cervical cancer?
Interviewee: No, I haven't.
Interviewer: Are you aware that ------provides cancer screening services?
Interviewee: I don't know if they offer screenings, but I know they provide treatment.
Interviewer: They do offer early detection services, and they're free. They provide free screenings for cervical cancer and breast cancer, and if you need to be checked for other issues, you can see specialists who will conduct thorough examinations.
Interviewee: So, there are services at ------for both breast and cervical cancer, and many health centers are currently providing these services.
Interviewer: Exactly.
Interviewee: I'd like to know how I can recognize if I have symptoms of cervical cancer.
Interviewer: One key symptom is irregular menstrual cycles; for example, if your period stops unexpectedly or comes early. Changes in your cycle, such as heavier or lighter bleeding than usual, indicate a need for screening. It's essential to get checked even if you don't have symptoms. Don't wait until you notice something is wrong; you should go for check-ups regularly.
Interviewee: We need sufficient education on this.
Interviewer: Absolutely.
Interviewee: Thank you.
Interviewer: It looks like we're done here
 
