# Supplementary material for: “If diagnosed early, you will be stressed and die…” drivers for breast cancer screening services uptake among women in Dar es Salaam
Source: PLOS Glob Public Health. 2024 Nov 4;4(11):e0003390. doi: 10.1371/journal.pgph.0003390 (PMC11534240; doi:10.1371/journal.pgph.0003390)
Supplement: S1 Data — (ZIP) [file pgph.0003390.s001.zip › TRANSCRIPT DATA EDITED/IDI OLDER WOMAN 03.docx]

**IDI-OLDER WOMAN 03**

**TIME: 22:46 MINUTES**

**TRANSCRIBER: ……….**

**Interviewer:** As I mentioned at the beginning, breast cancer is increasing among women, and many come when it is already too late. If a tumor is severely affected, it needs to be removed, although there was a chance it could have been detected and treated earlier. Now, we want to evaluate the effectiveness of messages or campaigns that encourage people to get preliminary checks before symptoms appear, especially for breast cancer. This is because it is essential for individuals to perform self-examinations correctly. So, we want to understand, when you came here for a breast cancer examination, where did you first hear about this service?

**Interviewee:** I went to a hospital near ----. When I arrived, I had a problem and was told, “You need to go there to get tested, that’s where the problem will be identified. But we cannot do it here.” I left and went there. When I arrived, they told me, “We have tests for cervical cancer, but we cannot perform them here due to the costs. You need to go to a government hospital, ------.” So, I started coming here. When I arrived and met with the doctors, I was well received. I asked as a newcomer. What year was this? When I came and had my examination, I was found to have a minor issue with cervical cancer, but no problem was found with my breasts. So, I left, attended to my issue, was given medication, and upon returning, I was told I was fine, but there was no issue with my breasts.

**Interviewer:** Ah! Have you ever heard anywhere about messages or announcements encouraging people to get checked?

**Interviewee:** Yes! Like on Clouds Radio, they announce a lot. And during that period, they came to---, and many people showed up for screening. I was also examined with that machine and was told I had no issues.

**Interviewer:** Okay, during the examination, did you feel that there was any important information you should have received before the examination?

**Interviewee:** I just wanted to know that I was okay. And I was thankful to be told that I was well.

**Interviewer:** When you were going for the examination, was there any education or lesson you received?

**Interviewee:** Yes! We received education from the nurses who examined us. We were educated about cervical cancer, breast cancer, hepatitis, and colorectal cancer.

**Interviewer:** What specifically about breast cancer did they talk about?

**Interviewee:** They said it is important for us to perform self-examinations at home. Additionally, while it can be related to women not breastfeeding or genetic factors with a high probability of occurrence, it is advised that women breastfeed their children to reduce the risk of breast cancer.

**Interviewer:** Hmm! Thank you very much. It seems you have a good memory. What is your opinion on the information you received? Perhaps what could be improved or added so that more people would be encouraged to get checked?

**Interviewee:** I tell women that it is better to get checked early, which is why I come here so that people get more motivation. Announcements should continue and, if possible, be made at homes so that someone might be told, “I’m going to ------,” and they would be surprised and think, “What are you going there for? You are going to look for a problem?” Many people refuse completely, not knowing they can get help if they come early. When you come, you might get treated and recover, as we see with others who have recovered.

**Interviewer:** Why do you think people fear going to ------?

**Interviewee:** They fear it! They think, “You have this cancer disease,” and become afraid. For example, if I tell someone I’m going to the hospital, they might say, “What are you going there for?” They believe that it’s a place where you only find serious diseases like cancer. They would prefer to stay home rather than seeking help, thinking it’s better to suffer silently than to find out about a problem.

**Interviewer:** Okay, so how can we improve these messages or campaigns?

**Interviewee:** Continue increasing the advertisements, perhaps through radio or by going into the streets. For example, where I live, there is a local government chairman. When you go there and talk to him during village meetings, you can discuss health issues, and it might raise awareness among people, encouraging them to get checked.

**Interviewer:** Thank you very much. Do you think people, even if they hear messages and announcements, will change their views on this issue?

**Interviewee:** Some people have a negative view. For example, at a parents' meeting at my child's school, there was a woman speaking about cancer. Some women mocked her, showing that acceptance varies just like in our communities where some accept while others do not.

**Interviewer:** How did people respond to the information they received?

**Interviewee:** Some think it’s just unnecessary pressure. They might say, “You’re just bringing us pressure to go to the hospital, but I don’t have money for treatment.” They might perceive it as an extra burden rather than seeing the benefits of early diagnosis.

**Interviewer:** So they feel the information does not concern them or adds to their stress?

**Interviewee:** Exactly. They feel it’s not relevant to them or adds to their stress, and they are more inclined to ignore it.

**Interviewer:** When you consider your close family, how do they perceive it when they know you’re going for a cancer screening?

**Interviewee:** For some, for example, when I told my sister, she was surprised and asked, “You’re going?” But others, like my sister who has had issues, understands the importance and encourages it, knowing the benefits of regular checks.

**Interviewer:** What about your other family members?

**Interviewee:** At home, my family is aware, but they are hesitant. For instance, one relative believes it’s better to suffer silently than to go for tests that might reveal a problem.

**Interviewer:** From your experience with this service, how would you describe the breast cancer screening service here?

**Interviewee:** I find it very good. The service has improved a lot. Initially, you would just get medication and leave, but now you meet with a doctor who prescribes medication and gives you detailed advice. The service has become more comprehensive and user-friendly.

**Interviewer:** Is there anything specific that you think should be added or improved in the breast cancer screening services?

**Interviewee:** I believe the service should continue as it is, as improvements have already been made. The introduction of specialist consultations and better organization of appointments has greatly enhanced the service.

**Interviewer:** Do you have any final questions or comments before we finish?

**Interviewee:** No, just that there should be continued encouragement and outreach, even in remote areas. Many people are unaware of the importance of screening and may only seek help when their condition is advanced. It’s crucial to reach out to as many people as possible so they understand the benefits of early detection and treatment.

**Interviewer:** Thank you. Have you reached menopause?

**Interviewee:** Yes!

**Interviewer:** In your opinion, at what age should someone start getting screened for cervical or breast cancer?

**Interviewee:** I think even starting at eighteen years old is good. Some young women, even as young as fifteen or eighteen, experience issues and should be checked.

**Interviewer:** Were you aware of the HPV vaccine for cervical cancer?

**Interviewee:** I had heard of it, but I wasn't very familiar with it. I saw some information about it at the school but didn’t know much about it until recently.

**Interviewer:** Have your children received the vaccine?

**Interviewee:** My child is still young, in the fifth grade, so not yet.

**Interviewer:** Thank you very much for your cooperation.

**Interviewee:** Thank you.

**Interviewer:** May God bless you.

**Interviewee:** Amen
