# Supplementary material for: “If diagnosed early, you will be stressed and die…” drivers for breast cancer screening services uptake among women in Dar es Salaam
Source: PLOS Glob Public Health. 2024 Nov 4;4(11):e0003390. doi: 10.1371/journal.pgph.0003390 (PMC11534240; doi:10.1371/journal.pgph.0003390)
Supplement: S1 Data — (ZIP) [file pgph.0003390.s001.zip › TRANSCRIPT DATA EDITED/IDI OLDER WOMAN 04 WAV.docx]

**IDI-OLDER WOMAN 04**

**TIME: 27:37 MINUTES**

**TRANSCRIBER: …………………**

**Interviewer:** So, the first thing we are looking at is where you first received the information about this examination, or what specifically made you come to get checked?

**Interviewee:** As I noticed… I had already completed my menstrual cycle. After that, I saw it, then I saw it again, and I noticed it about four times. I waited for about three years and saw it again, then waited for about two months and it returned. Since the eleventh month, it has recurred about three times consecutively. It’s not exactly consecutive but rather after seven or eight days, or after ten days or more. Then I became anxious, and this is the second month of this year that I came. I got this information saying there is treatment, that there are patients who have already been treated here, and I know that treatment is available here.

**Interviewer:** So, did you hear about this from patients, your relatives who have previously been treated here?

**Interviewee:** Yes!

**Interviewer:** Thank you very much. And what did you hear? What were they saying more? Did they give you any advice?

**Interviewee:** They told me that if you notice certain symptoms, you should come to ------. Some patients are even escorted, and you wait until you are overwhelmed. Moreover, even the nurses there write, “Please don’t wait until you see symptoms, like lumps in the breast, if you see something, come here first to get tested.” It is not necessary to wait for symptoms now; health check-ups are important. You should come for a check-up after some time without waiting to see symptoms, as it means you have already been affected.

**Interviewer:** Regarding the education you received, what else would you like to hear from the nurses or perhaps on TV? What would you like them to talk about?

**Interviewee:** They should discuss it so that I can hear. They talked about factors that trigger diseases, like young girls starting sexual activity early, having multiple partners. That’s what they talked about a lot.

**Interviewer:** What haven’t you heard them say?

**Interviewee:** I’ve heard most things except that storing alcohol and things like salt is not very good. For instance, if you have already cooked food and then start adding salt later, it is not good. Also, processed items like juices are better if you make them yourself. That’s what I understood along with many other things.

**Interviewer:** Before coming here, did you hear from your relatives who have suffered from cancer, or did you ever hear anywhere else about cancer screening?

**Interviewee:** I hear about it on the radio, and I hear it on TV; they talk about cancer issues.

**Interviewer:** From the information you heard, how would you like them to reach more people effectively?

**Interviewee:** For us women, if they could create lessons in schools for students from grade seven, form one, and beyond, it would be good. For grade seven students, even though they may start being curious, if they hear about it early and know it is dangerous, perhaps they would avoid it.

**Interviewer:** Okay. So, when you heard that there are treatments for cancer at ------, how did you initially take that information from your relatives or from TV?

**Interviewee:** I took it as if the disease is real. I took it seriously that the disease exists and the hospital provides treatment.

**Interviewer:** Did you think that message was about you?

**Interviewee:** Me!?

**Interviewer:** Yes.

**Interviewee:** I know it concerns me. I am a woman, and I know the disease affects me, but I wasn’t sure when it would come or if I would live until then or not. I was not sure about that.

**Interviewer:** How did you perceive ------ when you heard about it?

**Interviewee:** ------! How did I perceive it?

**Interviewer:** How did you perceive it, like, should I go to ------? What picture did you get before you actually came to ------ for treatment?

**Interviewee:** I perceived it like any other hospital. I didn’t find it alarming. Regarding the disease, I knew that if I missed this one, another one would find me. That’s how I perceived it. Even if I have the disease, I thank God and accept the results. It’s not that I like it, but I have to accept the results if I am already ill. I will not just sit there in worry about the disease. I will see the doctor and let them treat me.

**Interviewer:** When you look at your close ones—your children, neighbors, people in your community—how do they perceive breast cancer screening?

**Interviewee:** You know, they find it frightening! They view cancer screening as something alarming. Even so, I’ve shared it with my neighbors, and I fear that it might alarm them.

**Interviewer:** So you haven’t told them…?

**Interviewee:** I haven’t told my neighbors, but I have told my children. I have informed them that I am ill, and once I get the results, like if they say “Mom, you have cancer,” I will tell them. But I haven’t told my mother; I feel she might worry a lot if I tell her. However, I am looking for a chance to tell her because if she hears from someone else without me telling her, she might feel bad. But I have told my children and some close relatives that I am going to the hospital.

**Interviewer:** How do they receive that information?

**Interviewee:** My children have received it. They understand, but they are not entirely satisfied. They can’t be satisfied because when a person says they are ill, it’s hard for others to feel good about it. But they have to accept the results.

**Interviewer:** And have you told your female children that it also concerns them?

**Interviewee:** Yes, I told one of them directly, but unfortunately, she was pregnant, and I hear that pregnancy might not be advised. Yes, since she was pregnant, I planned to bring her, but it was three months ago, and unfortunately, it was as it was. Now, one of my younger siblings said, “No, no!” and wanted to accompany me. Unfortunately or fortunately, I didn’t tell her when I was coming, but she wanted to accompany me so she could also get tested after being guided. Here, pregnant women are not advised to come; one should start seeking help early to avoid complications. But if you have already suffered, it’s difficult; you recover with God’s help.

**Interviewer:** But how do others perceive cancer? When they hear about cancer screening or someone having cancer, what is their perception?

**Interviewee:** They perceive it as a death sentence.

**Interviewer:** What do they say is the cause in the community?

**Interviewee:** In the community, I haven’t heard about the cause of cancer, but I came to learn about it here. For example, some believe that a woman who is still a virgin is less likely to get the disease, and if she does, it might be in other areas like the breast.

**Interviewer:** For breast cancer, what do people believe is the cause? Before you came to learn about it here, had you heard anything?

**Interviewee:** Honestly, I didn’t know; I thought it just starts. But here I learned that it can be hereditary, that consuming fatty foods, smoking, and other factors contribute. Initially, I didn’t know what it was related to; I thought it just starts. Maybe you can clarify if I’m correct.

**Interviewer:** I’m asking you because I want to know from you.

**Interviewee:** So, you are my student?

**Interviewer:** Yes. I know you know more than I do.

**Interviewee:** Do I know? You’ve studied. I haven’t studied.

**Interviewer:** I want to know from you, even if you haven’t studied; you may know more, and learning has no end.

**Interviewee:** As we are told, education has no end. As I mentioned, I learned this as an adult. I didn’t know it in my youth or childhood; I only learned it recently. For that reason, I now share it with my children and will tell them, “This and that can cause problems. Having many partners can lead to disease.” They also say that viruses are transmitted by men, but if men bring them and your immunity is good, those pathogens usually disappear. However, if your immunity drops, that’s why at a certain age, those pathogens become present.

**Interviewer:** Thank you very much. Now, you mentioned that you have heard messages on TV and radio about raising awareness. Have you heard them discussing anything specific?

**Interviewee:** Oh! I am usually very lazy to listen, except that I hear “Let’s go for breast screening; there is breast cancer.” But I don’t listen closely or follow up.

**Interviewer:** If you’re not following up on the radio, what other methods could be used to reach people who are busy with their own affairs to get this message?

**Interviewee:** For this message, if I could get it…!

**Interviewer:** If you want to reach someone effectively, what should be done?

**Interviewee:** It’s good on TV. Many people watch TV; I watch a lot of TV myself. Most people watch TV, so that’s effective.

**Interviewer:** What about those who don’t have a TV?

**Interviewee:** There are books available. Small booklets could be made for women, maybe distributed, like if they reach out to local government representatives. If they receive these booklets, someone who wants to read could get the message effectively.

**Interviewer:** What are your thoughts on the educational announcements you have heard on TV and radio? Do you think they are effective?

**Interviewee:** They are effective for those who listen. Some people, if they can watch or listen, might act upon it.

**Interviewer:** How would you describe the information or education you received here compared to what you’ve heard elsewhere?

**Interviewee:** The education I received here was good and useful, unlike what I’ve heard elsewhere. I came to understand it better here.

**Interviewer:** Okay, thank you very much.

**Interviewee:** Thank you
