# Supplementary material for: “If diagnosed early, you will be stressed and die…” drivers for breast cancer screening services uptake among women in Dar es Salaam
Source: PLOS Glob Public Health. 2024 Nov 4;4(11):e0003390. doi: 10.1371/journal.pgph.0003390 (PMC11534240; doi:10.1371/journal.pgph.0003390)
Supplement: S1 Data — (ZIP) [file pgph.0003390.s001.zip › TRANSCRIPT DATA EDITED/IDI YOUNG WOMAN 03.docx]

**IDI-YOUNG WOMAN 03…**

**TIME: 30:31 MINUTES**

**TRANSCRIBER:…………..**

**Interviewer:** We are reviewing evaluations of reports related to investigations for at-risk clients. We want to know, for the very first time, how you came here for a breast cancer examination. How did you find out about this service and where is it available? Where did you get the information?

**Respondent:** Ah! First, before I got the information, there was something unusual happening in my body. When I was married but had not yet had a child, I started experiencing pain in my breasts. So, this pain made me start inquiring because a friend of mine was studying to be a surgeon, so she gave me the information. I asked her why I was feeling this way. She told me to go to the hospital, to go to ------, and that’s how I became aware. I found out that ------is for cancer screenings. She explained that they conduct cancer tests. That was in 2013. But after asking her, I was still feeling pain, especially since I was married and had just started experiencing small pains about a month into my marriage. The pain in my breasts made me question what was happening. Why was it hurting? She told me to go to ------. But before going to ------, that friend advised me to do a small check-up first at ------, then go to ------. I did the check-up and had a mammogram, which came back clear. However, at that time, I was pregnant and did not realize it. I was about two months pregnant. But they did not tell me or test for pregnancy, so when I left, I was still in pain. They told me I was okay, but I still felt pain. I told my husband that I thought I should go to ------since I still didn’t understand my condition. I came here again and had another check-up (speaker announces loudly). After two weeks, I started vomiting and realized that I was indeed pregnant. I went to the hospital and then returned to ------. They confirmed that the lump was due to hormonal changes because milk production had started. That’s when I stopped the whole process because I had reassured myself that I was okay after going to ------. I gave birth to my first child and after about three years, I conceived again. But when I conceived, I was not aware of the pregnancy. I didn’t take it seriously; I thought I was safe. But it turned out I was pregnant. With the second pregnancy, I again started experiencing painful breasts, pain so intense that I felt burning and cried at night. My husband was surprised and asked, "What is this?" I was in distress, saying it was unbearable. At that time, my husband had been transferred to ------, so I went to ------ Hospital but they did not find anything. My husband told me, “No, there’s nothing. Go to Morogoro.” I was referred to Morogoro city. I went and had a check-up, and they told me, “Return to Dar es Salaam. The whole time, my dear, I had no idea it was cancer.” I returned to Dar es Salaam and came to ------for the second time. I had another check-up and was told, “There’s nothing.” But the second time, I was in agony, especially on the left side. Today, I came back because I still felt pain, like a boil when pressed. The pain persists, and sometimes when I wake up, I feel okay. I’ve realized that even in the last two or three days, even when I wear a pad and bend over, I feel pain. So, I went for a check-up and was told I was fine. I stayed pregnant for about two months, and I thought, “Maybe this is normal at this stage; my first symptom was pain.” I gave birth to my child, who is now three years old. I thank God for the second chance. I stayed for a week today, and the pain started again. My breasts began hurting, and I was supposed to have my period on the 15th. My husband is a government employee in ……, so I stayed with him for the past week. But now, I feel like my breasts are hurting. My husband advised me to return to ------, suggesting it might be a pregnancy but I still don't know. The nurse advised me to wait until the 15th of the upcoming month; it will be a month since then. So, if I don’t get my period, I will know if I’m pregnant. Then, after giving birth, if the child is four months old, I can come back for an examination.

**Interviewer:** Okay. So, you learned about ------through experiencing pain?

**Respondent:** Yes! Through the pain.

**Interviewer:** After that, have you ever come across any information or campaigns encouraging people to get screened?

**Respondent:** I have come across them. My sister, a classmate, and I studied together. She was diagnosed with cancer in----. We finished school in …., and she was diagnosed with cancer in 2014. She is very close to me, and we used to talk a lot. She was a grown woman who was diagnosed with cancer in----. She told me, “You know, my sister, I went for a check-up and was diagnosed with cancer.” This made me very aware of these issues. Whenever I have a problem, I come here.

**Interviewer:** Ah! Regarding the awareness campaigns for cancer screening, have you ever seen such campaigns on the radio or TV?

**Respondent:** I hear a lot about them. Nowadays, I think the media plays a significant role in disseminating information. We see doctors going to ….. and different regions. They show these on news reports. It’s not like before; I think there is now a greater awareness compared to the past.

**Interviewer:** How do you find the information that is broadcast? Is it adequate or does it need more content to encourage people to come for screening?

**Respondent:** Actually, it helps a lot, especially for us women in the community. Because you find that women are the primary caretakers and they are often busy, some are single mothers. So, the time to come for a check-up is limited because they are struggling with life, worrying about what their children will eat if they don’t do this or that. Therefore, the information is genuinely helpful.

**Interviewer:** When you came for your examination, had you ever received any education or training on this?

**Respondent:** No, I hadn’t. I came in 2013, and I had never received any education on this. I just had the examination and was told I was okay.

**Interviewer:** Regarding our current method of distributing information—through radio, TV, and doctors conducting campaigns—do you have any opinions on this? What should be done to encourage more people to come?

**Respondent:** My opinion is that, for example, those involved in blood donation often set up camps in different places, such as entertainment events where people donate blood. I think for breast cancer screening, you should also do something similar. Set up camps, provide education, even using loudspeakers on the streets. Especially for many women, the difficulty of life contributes to the situation where they are unwell but do not know that there is free service available. They lack someone to push them; they worry about “What will I eat, what will my children eat?” So, you should try setting up camps, similar to what those blood donation organizations do. They make an effort and attract people. People gather to see what’s happening. Therefore, I recommend you try that approach.

**Interviewer:** Thank you. Let’s talk about perceptions now. How do you feel about the awareness of breast cancer screening? How do you feel when you see these advertisements?

**Respondent:** First, what attracts me the most is that I have had two or three friends with whom we discussed cancer screening. One of them said, “And it’s free! So why don’t women go? The screening is free?” When you go to the hospital, you have to pay for regular services, but women still don’t show up. So many people think, “This thing is advertised as free, but why is the response so fearful?” People have developed fear. But for me, when I heard the term “free of charge,” I was encouraged. I felt hopeful and thought that even if I heard it from a friend or relative, it would be easy to go. You know, my dear, life has become very hard, so if you tell people it’s free, many Tanzanians are attracted. It’s good because it raises awareness.

**Interviewer:** How do people close to you perceive the idea of preliminary cancer screening? If someone has no symptoms and you suggest a screening, how might they respond?

**Respondent:** I just spoke with my sister recently and told her I was at ------. She said, “Oh! I’m scared.” So many people have fear. Women, in particular, still have fear.

**Interviewer:** What are they specifically afraid of?

**Respondent:** The results, yes! The word “you have it” makes them feel as if... most people are very scared.

**Interviewer:** Let’s discuss the reception of services. How did you find the service?

**Respondent:** The service today is very good. I haven’t stayed long. I found people and didn’t stay long. I was received well; the nurse received me well. The doctor also received me well and provided clear answers. The service is truly good.

**Interviewer:** Thank you. Do you feel the service is beneficial?

**Respondent:** YES! It is very beneficial. It is beneficial because first, when you do the examination, you feel

Certainly! Here is the translation from Swahili to English, paragraph by paragraph:

**Interviewer:** We are looking at an evaluation of reports involving investigations for at-risk clients. We want to know, when you first came here for a breast cancer examination, where did you get the information that such a service exists and where it is available? Where did you get the information?

**Interviewee:** Ah! First, before getting the information, there was something I felt was different in my body. Before I had children, when I was married, I started feeling pain in my breasts. So, that pain made me start asking around because a friend of mine was studying to become a surgeon, so she was the one who gave me the information. I asked her why I was feeling this way. She told me to go to the hospital, to go to ------. That’s how I became aware, oh! I didn’t know that ------was for cancer testing. She told me that they test for cancer there. That was my awareness in 2013. But after asking her, I was still feeling pain. I was married and about a month into the marriage, I started feeling minor pains. But now the breasts were hurting and I was wondering what this was. Why are they hurting? She told me, “Go to ------.” But before going to ------, that friend advised me to go to ------ for a small check-up before heading to ------. I did the check-up and had a mammogram. They said, “No problem.” But at that time, I was pregnant and didn’t know I was pregnant; I was about two months pregnant. But they didn’t tell me, they didn’t detect the pregnancy. So, I left there still feeling pain, and they told me, “You’re okay,” but I still felt pain. I told my husband I thought I should go to ------because I still didn’t understand this condition. So, I came here, and they also did a check-up (the speaker announces loudly). After two weeks, I started vomiting, and I realized, oh! This is already a pregnancy, so I went to the hospital. I went back to ------, and they told me, “This lump is due to changes because it is caused by milk production.” That’s when I stopped all the processes because I was already assured that I was fine after going to ------. So, I left it. I gave birth to my first child and stayed for about three years, then conceived again. But when I conceived, I thought it was a pregnant. I wasn’t paying attention to the fact that it was a pregnancy. Mmm! Because I thought, oh! You know, that trick where you think you’re safe, but it looked like I am pregnant again. I it started the second time, and now the breasts was so painful that I felt like it was burning, and I cried at night. My husband was surprised, “What is this?” I cried and said, no, this is not possible, what is this? At that time, my husband had been transferred to ------, so I went to the ------ hospital, and they didn’t find anything. My husband told me, “No, there’s nothing. Go to the Morogoro hospital.” They referred me to Morogoro, in the city of Morogoro. I went and had a check-up, and they told me, “Return to Dar es Salaam.” During all this time, my dear, I had no idea it was cancer. I returned to Dar and came again to ------. This is my second time. I came, had a check-up, and they told me, “There’s nothing.” But the second time, I was even crying, feeling severe pain, especially here on the left side. And today I returned because I am feeling pain, like a boil when I press. I feel pain; sometimes when I wake up, I feel okay. I’ve noticed in these last two or three days, even when I wear a pad, and bend down like this, I feel pain. So, I went and had a check-up, and they told me I’m fine. I stayed for about two months of pregnancy, and I thought, oh! Maybe it’s normal at this stage; my first symptom was pain. I gave birth to my child, who is now three years old, and I thank God for the second time. I’ve stayed like a week today, and it seems like the pain has started again. But the breasts have started to hurt; my period is supposed to start on the…. My husband is a government employee in Dodoma, so I stayed for the past week. But now, why do I feel like the breasts are hurting, like my body? When I press like this, my husband told me again, “Return to ------. Maybe you’re saying it’s a pregnancy, but it’s not possible. Why do you have symptoms like these, like boils? Why?” So, my dear, that’s why I’ve returned today. I’ve come for a check-up and have been told I’m okay. I told that lady that maybe I’ve conceived because it’s been about a week. Even if we go for a test, they won’t be able to tell if the person is pregnant. So, the nurse advised me and said, “Stay at least until the 15th of the coming month. That will be one month. So, if your period doesn’t come, you will know you’ve conceived. Then, after giving birth when the baby is four months old, you can come back for a check-up.”

**Interviewer:** Okay. So, did you find out about ------through pain?

**Interviewee:** Yes! Through pain.

**Interviewer:** After that, have you ever come across any information or campaigns encouraging people to get screened?

**Interviewee:** I have come across them. My sister, a classmate, and I all graduated from university. She was diagnosed with cancer in…., after we finished our studies in ….. She is a very close person with whom we used to talk and share stories. She is an older person who was diagnosed with cancer in …. and came to tell me, “You know, my younger sister, I went for a check-up and was diagnosed with cancer.” So, through her, it made me very aware of these things. When I have a minor problem, I come here.

**Interviewer:** Ah! Speaking of awareness campaigns. Have you ever seen such campaigns on the radio or TV?

**Interviewee:** I hear a lot about them. Nowadays, I think the media is very helpful in providing information. We even see doctors going to …. and other different regions. They show this on news reports. Indeed, nowadays it’s not like before. I think people’s understanding has increased compared to previous years.

**Interviewer:** How do you view the information provided in these reports? Do you think it is sufficient, or does it have content that would encourage people to come for screening?

**Interviewee:** Honestly, it helps a lot, especially for us women in the community. Because you find that women are the main caregivers, they are busy, and some are single mothers. So, the time to say, “Come for a check-up” is limited because they are struggling with life and thinking, “If I don’t do this, what will my children eat?” So, it really helps.

**Interviewer:** When you came for a check-up, had you ever received any training or education on this?

**Interviewee:** No, I hadn’t. I came in 2013, and I hadn’t received any. It was just that I went for a check-up and they told me I was okay.

**Interviewer:** Regarding our current information dissemination system, including radio, TV, and doctors’ campaigns, do you have any suggestions on how to improve so that more people come?

**Interviewee:** My suggestion is that, for example, the Blood Bank people set up camps… like where we are, they come a lot, and you find they organize entertainment and people donate blood. I think for breast cancer screening, you could also do that. Like organizing some camps with entertainment and providing education even with loudspeakers on the streets. Especially for many women, I think the difficulty of life contributes, and you find that others in our community are indeed suffering but don’t know it’s free service. But people need a reminder, they don’t have someone to push them. They think, “If I go, what will I eat, what will my children eat?” So, I suggest you also set up camps like they do at the Blood Bank. They try hard. Go to different places with some entertainment. People gather to find out what’s happening. So, I suggest you try that.

**Interviewer:** Thank you. Let’s talk about perceptions now. What are the perceptions of awareness campaigns for breast cancer screening? When you see such advertisements, what feelings do you get?

**Interviewee:** First, what attracts me is that I have had discussions with two or three friends about cancer screening. One said, “And it’s free! I didn’t know it was free, but why don’t women go? But the screening is free?” When you go to the hospital for regular services, you contribute, but women don’t show up.” So, many people think… they have a perception that “this thing is advertised and it’s free, but why are the responses so fearful?” People have built up fear. But for me, you know, I was motivated when I heard the word “free of charge,” no cost at all. I was encouraged. I said, “Even if I hear from a friend or relative, it’s very easy.” You know, my dear, for me, it wasn’t something scary. But people, I think, have fears, and they say it’s free, but they are afraid. They haven’t built up the courage. But if I were to take a friend or tell her to go, I would just say it’s free and encourage her to go. I was never afraid. Even when I came for a check-up, I saw that it was free. I didn’t pay any extra, and it’s free service.

**Interviewer:** For those who are afraid or haven’t gone for screening, what are their main concerns?

**Interviewee:** Their main concern is that they think it’s a costly thing or that it involves a lot of procedures and they fear the diagnosis. I see that’s why some people are afraid. They say, “No, no, no! I’m not going because if I go, they will find something, and the treatment will be very expensive. It’s better to stay in my situation than to go and find out.” So, fear is very high. Others say, “Even though it’s free, the treatment may be very expensive, and I might not afford it.” So, fear is high.

**Interviewer:** Thank you very much for your time.

**Interviewee:** You’re welcome. Thank you very much.
