# Supplementary material for: “If diagnosed early, you will be stressed and die…” drivers for breast cancer screening services uptake among women in Dar es Salaam
Source: PLOS Glob Public Health. 2024 Nov 4;4(11):e0003390. doi: 10.1371/journal.pgph.0003390 (PMC11534240; doi:10.1371/journal.pgph.0003390)
Supplement: S1 Data — (ZIP) [file pgph.0003390.s001.zip › TRANSCRIPT DATA EDITED/IIDI OLDER WOMAN 04.docx]

**IDI-OLDER WOMAN 03**

**TIME: 22:46 MINUTES**

**TRANSCRIBER: ------.**

**Interviewer:** As I mentioned earlier, breast cancer is increasing among women, and many come for screening when it's too late. When the tumor is significantly affected, it needs to be removed, although there was a chance it could have been detected and treated earlier. Now we want to assess the information or messages from campaigns encouraging people to seek early screening, especially for breast cancer, because the way one examines themselves can reveal issues. So, we want to know, before you came for your breast cancer screening, where did you get the information about this service?

**Interviewee:** I went to a hospital near -----. When I arrived, I had a problem and was told, “You need to go there for tests to find out what the issue is, but we cannot do it here.” So, I left and went to that place. Upon arriving, they told me, “We can do cervical screening, but we can’t proceed because it’s expensive here. So, go to the government hospital, ------.” That’s how I came here. When I arrived and met the doctors, I was welcomed well. I asked questions because I was a newcomer. What year was this? When I came, I was examined and found to have a minor issue with my cervix, but there was no problem with my breasts. So, I left and focused on my issue, was given medication, and when I returned, I was told I was fine, but I was okay regarding my breasts.

**Interviewer:** Have you ever heard any information or messages encouraging people to get screened?

**Interviewee:** Yes! Like on Clouds Radio, they announce a lot. They also had a program, and when I went there, many people showed up, and we got screened. I was called in again, and they used that machine, telling me I had no issues.

**Interviewer:** Okay, during the examination, what important information would you have liked to receive before being screened?

**Interviewee:** I just wanted to hear that I was fine. I was grateful when I was told I was okay.

**Interviewer:** When you went for the screening, were you given any education or information?

**Interviewee:** Yes! We were first educated by the nurses who examined us. We were informed about cervical cancer, breast cancer, hepatitis, and colorectal cancer.

**Interviewer:** What did they say about breast cancer?

**Interviewee:** They said it's important for us to examine ourselves at home. They also mentioned that not breastfeeding children can increase the risk of breast cancer, especially in families with a genetic predisposition. So, it’s advised that women try to breastfeed their children as it reduces the risk of breast cancer.

**Interviewer:** Thank you very much. It seems you have a good memory. What are your thoughts on the information you received? What could be improved or better communicated so that more people would go for screenings?

**Interviewee:** I tell women that it’s better to get screened early, and that’s why I come here to encourage more people. Advertisements should continue to be made, and if possible, spread in homes. You might find someone saying, “I’m going to ------,” and they react, “What? You’re going to do what? You’re looking for something serious? I’m not going.” People refuse to go, but if you come here, you get help; you realize your condition early. If it’s still minor, you can be treated and recover, as we see others recovering.

**Interviewer:** Why do you think people fear hearing “------”?

**Interviewee:** Yes, they fear! They say, “You have this cancer disease,” and they become scared. If I tell someone I’m going there, they might not even ask where. They react with shock, thinking, “Why are you going there? That place has those diseases, you’re going to search for something?” They’d rather stay home than find out. You find neighbors like that; I don’t tell them where I’m going because they panic.

**Interviewer:** Okay. Regarding improving this information or campaigns, what do you suggest we do?

**Interviewee:** We should continue to put out more announcements, if possible…

**Interviewer:** What kind of announcements?

**Interviewee:** On the radio, or if possible, going door-to-door. For example, I live in a certain area. When you arrive there, you can meet the village leaders. When you explain during community meetings, you can reach people, and maybe one person will say, “Let me also go get screened.”

**Interviewer:** Thank you very much. Do you think people would listen to you if you stood there and spoke?

**Interviewee:** Some might not have a good perspective. For instance, my child studies at -----, and during a parents' meeting, a lady talked about cancer issues. Some mothers laughed at her, you know? It’s like our prophets; some accept them while others reject them. When that lady spoke, some teachers listened, but others were dismissive. However, that’s human nature; people vary in their responses.

**Interviewer:** How did people feel after receiving that information? What was their reaction?

**Interviewee:** Some felt like we’re just bringing pressure to them! One might say, “You’re just giving us pressure so we can go to the hospital, and I don’t have money for treatment.” Some may think that way, “You’ll die from pressure while knowing you have no money for treatment.”

**Interviewer:** So they view this information as not applicable to them? Does it stress them out because it concerns them?

**Respondent:** When we are the ones who should be getting checked!

**Interviewer:** Hmm! Thank you very much. When your close family at home knows that you’re going for a cancer screening, how do they react?

**Respondent:** For some, like if I tell my sister, she gets surprised and says, “You’re going?” But there’s another sister of mine who has issues, you see? (Interviewer: Hmm!) She got infections, and they say that every time she goes, she must be checked. So she knows it’s very important and tells me, “It’s really good because you need to get checked.” But for the others, you find that they are filled with fear.

**Interviewer:** How about your peers at home?

**Respondent:** At home? (Interviewer: Hmm? Have you told her?) Hmm! I tell her I’m going, but she’s resistant.

**Interviewer:** What’s her perspective?

**Respondent:** She thinks, “Ah! You’re going, go ahead.” She doesn’t see it as important or necessary. I have to do it myself; I take care of it myself. But even when I tell her that I’m taking medication during that time when she has issues, she still needs to take it. (Interviewer: Hmm!) When I come here and get the medication, I tell her she needs to use it when I get back, but she says she doesn’t want to. You see? Sometimes she’ll say, “Buy it for me; I don’t have money.” If you buy it, she might take it. They can be very resistant. Even for screenings, they tell us that men should be checked for prostate issues, and there’s no other way except through blood tests. So, please encourage them when you get there. She thinks it’s better for her to struggle with life than to face this. If you explain it to her, I tell her these issues exist and that she needs to be aware of them.

**Interviewer:** Okay! Now let’s look at the reception of cancer services. You mentioned that there was a time they had come for screenings. How do you think people received that screening service?

**Respondent:** People came in large numbers, truly. Yes, they received it well. And I had already come here before; I came, and then I was told to come back after a year, then two years to come again. So those who screened me here, I saw them there, and people really responded. Some even couldn’t get screened because the time was up. The nurses here directed us, saying, “Please, go to ------; we’ve reached our limit here,” because it was already around twelve, heading toward one. But people really filled up at the screening location.

**Interviewer:** What about those deep inside the neighborhoods?

**Respondent:** Hmm! I only met one woman. But over there, I mean, that’s why I say there’s still a need for more encouragement—people have become very resistant.

**Interviewer:** Personally, what benefits do you think this service offers?

**Respondent:** This service?

**Interviewer:** Yes, for early screening? For cancer?
**Respondent:** I really see it as very beneficial. Because when you come and are identified early, as they say, there’s that initial stage (Interviewer: Hmm!). So, when it’s detected early, it doesn’t become so severe, and you incur less cost. But when it’s late, you face much larger expenses and treatment. There was a woman who came here, and I think she’s from around here; I heard her encouraging people. She said, “I had it myself, but I’m grateful I have recovered.” Many people, even the day we arrived at the screening, a doctor came and stood up, saying, “You see this woman? She had colon cancer, but look, she’s standing. She caught it early. You can survive cancer if you catch it early, so don’t be afraid to come for screening.”
**Interviewer:** When we look at religious or government leaders, what kind of stance or influence do you think they could have to encourage people to receive cancer screening services?
**Respondent:** Over there, I’m not sure; maybe they haven’t been reached? (Interviewer: Ah!) Yes, perhaps if you reach them, they will be supportive. (Interviewer: Okay, can they influence people?) They can be influential.
**Interviewer:** They can influence their people?
**Respondent:** Yes! Because I feel that they haven’t been reached yet. But when they are reached, they will further encourage the people there, and they will take action.
**Interviewer:** Do you feel there’s a specific perception that has developed in the community regarding cancer or breast cancer screening?

**Respondent:** Are you asking about hospitals or in the community?
**Interviewer:** In the community, what do you think people think? When they hear that someone has gone to that hospital for a cancer screening, how do you think they perceive it?
**Respondent:** They perceive you as already sick, like you have a serious illness, and they think you’re going to die. You’ll find that when they see someone going to the hospital, they know it’s likely cancer. Instead of seeking help, they might turn to traditional healers, thinking they've been cursed when they haven't. For instance, there's a woman from a rural valley who, let’s say, was farming and was first diagnosed with an issue. My brother-in-law has married three women. So, this woman developed the notion that... but we who know (Interviewer: That notion, you mean she thinks she’s cursed?) Yes! We tell her it’s better to go get tested to understand what the issue is. If you see blood coming irregularly, you should get tested; it could be cancer. She went to one hospital, and when she arrived, they told her, “We can’t help you; go to another hospital.” After being referred, it was confirmed she had an issue, and she came to stay with our sister. She was truly thankful, even attending prayer gatherings, saying they treated her well during her screening. Initially, she was examined, and after her diagnosis, she was prescribed medication. Though the treatment was tough, we brought her fruits and vegetables according to the advice she received. Eventually, she recovered and returned home, continuing her life with scheduled follow-ups. This teaches you that when my sister was ill and got better, it’s better to catch it early to receive treatment quickly.
**Interviewer:** How did that situation with your sister motivate you?
**Respondent:** Yes! When that sister came, I had already been here (Interviewer: Ah!). I had already started my own screening. So I told her, don’t worry; we were encouraging her not to think negatively. Before she came to be tested, we assured her she would recover, and truly, God helped her get well.
**Interviewer:** Looking at everyone involved in helping your sister until she was treated and healed, how do you see their perception of these services now? What about your relatives?
**Respondent:** They…
**Interviewer:** How do they perceive it? Have they been screened?
**Respondent:** Yes!
**Interviewer:** Have they been screened? Or how do they view the issue of cancer?
**Respondent:** They recognize it exists, and they are getting screened. They are undergoing tests. For instance, that sister in ----- who was with the patient has undergone tests herself; everyone has been tested.
**Interviewer:** Ah! Now, looking at your experience with these services every time you come, how would you describe the cancer screening services here?
**Respondent:** Ah! I see it as good; yes, it’s good. When I first came, I was given medication, and now I’ve been told I need to meet with a doctor first because they need to examine me properly.
**Interviewer:** When we consider the people coming for screenings, what specific factors encourage or hinder them from seeking screening services?
**Respondent:** The people coming?
**Interviewer:** Yes, your close ones, those who don’t come—what holds them back?
**Respondent:** What holds them back is that they might think, “If I go there, everyone will know!” Some might say, “I’d rather suffer and die than go get tested and die from stress.” Others might think, “If it’s discovered I have an issue, I might die from stress!”
**Interviewer:** Wow! That’s true. And what about you? What motivates you and your peers to get tested for cancer?
**Respondent:** I’d say it’s better to get tested early to know and receive treatment because when treated early, you experience the benefits of that treatment.
**Interviewer:** When you arrive here, what impresses you the most?
**Respondent:** I’m impressed by their services. Their services are really good and well-organized. If you’re told you’ll meet a doctor, you will indeed meet a doctor who will write you a prescription.
**Interviewer:** And how long do you wait for the services?
**Respondent:** It’s not a very long wait. You arrive on time and are served promptly.
**Interviewer:** And how far do you travel from home to access these services?
**Respondent:** It’s far because I tell you I leave home at ten o’clock. Sometimes I’ve left home at nine in the evening and arrived here at night, waiting until twelve when people start to arrive. If I reach there around eleven, at twelve people start to show up. However, arriving early ensures I’m served first; that’s why I prefer to come early.
**Interviewer:** That’s good. What are your thoughts on these services? What should be done in breast cancer screening services? Specifically, what should be added or improved?
**Respondent:** I think you should continue as you are. Because currently, you’ve improved the services significantly. In the past, there were no doctors available, so when you came, you would just be given medication by a nurse and leave. But now you’ve improved the process; when you leave there, you’re told to see a doctor who will prescribe medication, and you can go buy it to use at home. The service has truly become easier, and I ask you to continue improving it further. Because now, I’ve realized I can meet a specialist; I didn’t know that until today, so you’ve enhanced the services.
**Interviewer:** What do you think should be added? Just as you mentioned? (Respondent: Yes!). Do you have any questions before we finish? Any other thoughts we haven’t discussed?
**Respondent:** No, just my thought is to continue encouraging people, especially in -----. You’ll find that some are suffering but don’t know; as I mentioned, people come in when the issue has become serious. If possible, try to reach those people so they can be informed that getting tested is a normal thing, and if an issue is found, they can quickly get treatment and recover.
**Interviewer:** Thank you. Have you completed menopause?
**Respondent:** Yes!
**Interviewer:** Ah! As you know, what age should someone start being screened for cervical or breast cancer?
**Respondent:** I think even at eighteen, they should be screened. Because you might find a young girl at eighteen or even fifteen who has that issue. She might say, “My breast hurts.” So starting at that age is not a problem. Now, since we’ve been informed about the cervical cancer vaccine, I asked the young girls I saw.
**Interviewer:** You didn’t know?
**Respondent:** I was only aware of the coconut palm. But when we came here, they mentioned the vaccine up to the coconut palm. However, one of my sister’s children was vaccinated at school, secondary school. That’s when they began giving the vaccine while distributing flyers.
**Interviewer:** Have your children received that vaccine?
**Respondent:** My child hasn’t! She’s still young, just in fifth grade.
**Interviewer:** Well, thank you very much for your cooperation.
**Respondent:** Thank you.
**Interviewer:** God bless you.
**Respondent:** Amen.
