# Supplementary material for: “If diagnosed early, you will be stressed and die…” drivers for breast cancer screening services uptake among women in Dar es Salaam
Source: PLOS Glob Public Health. 2024 Nov 4;4(11):e0003390. doi: 10.1371/journal.pgph.0003390 (PMC11534240; doi:10.1371/journal.pgph.0003390)
Supplement: Attachment — Submitted filename: Response to Reviewers.doc [file pgph.0003390.s002.doc]

Response to Reviewers Comments August 2024

Below are the response to your recent comments on our Manuscript

| **EDITOR's comments to the authors:** | **Response** | **Page number** |
| --- | --- | --- |
| No comment | We have revised the title to read, “**If diagnosed early, you will be stressed and die…” Drivers for uptake of breast cancer screening services among women in Dar es Salaam**. | Page no 1 |
| Data Collection Methods - The author should provide more information about some of the specific questions listed in the question guide. | Thank you very much, authors have revised the section and specific questions that were asked to elicit information on drivers for uptake of breast cancer screening services. | Page No. 7 |
| The subtitle 'methodological consideration' in the material and methods section should be revised to reliability and validity measures. | Thank you for an insightful comment, authors have renamed this section “reliability and validity” as suggested and the limitations have been moved to after the discussion part. | Page No. 9 |
| Ensure that the manuscript is revised in accordance with the author's guidelines and that the appropriate checklist is completed (see https://journals.plos.org/globalpublichealth/s/submission-guidelines#loc-materials-and-methods) | Thank you very much, We have revised and checked the manuscript and it adheres to the journals guideline |  |
| Results- Ensuring that the study's results are clearly presented and linked back to the research objectives will help readers understand the significance of the findings. A well-structured presentation of findings can help the research have a greater impact. As an example, there is a disconnect on how individual-level factors relate to indirect cost, distance to clinic.... You can keep it as it is but provide a linking narratives for the readers. Also, awareness of SBE or CBE and myths can each be under a sub-category - knowledge. Overall, for the results section, provide proper narrative linking the emerged themes to the figure 1 designed and how domains in the SEM model was covered. | Thank you for the comment, The results have been linked to the objectives of the study, Narrations have been added on how the indirect costs and distance to clinic are linked to individual level factors.  The subcategory on awareness and myth have been merged to form Knowledge category | Page No. 11-14 |
| Before the conclusion and recommendation section, It would be beneficial to include a section discussing the study's limitations. Addressing potential biases, data collection limitations, or other factors that may have influenced the results will improve the overall research. | Thank you very much for this insighful input. Authors have added a section to describe study limitations and measures applied to address. | Page No. 24 |
| Recommendations for Future Research: Identifying areas for future research on breast cancer screening drivers in various contexts or populations could help to deepen the study. Proposing new avenues of research can help to advance the field of study. | Thank you for Comment, Area for further research have been added | Page No. 24 |
| Lastly, consider grammar editing to make sure there are no errors and provide an English translated transcripts. | Thank you for this comment: We have edited the document and corrected the grammar errors in the whole document |  |
|  |  |  |
| **REVIEWER # 2 COMMENT** |  |  |
| Authors should discuss policy implications based on the study findings. Example, this study revealed that many women lacked awareness about breast cancer, its symptoms, the importance of screening, and the benefits of its early detection. What strategies can address these knowledge gaps? (You may consider educational campaigns utilizing various channels such as radio, television, community meetings, and educational materials in the local language to reach a wider audience). Additionally, the study reported that the financial burden (transportation and time off work) emerged as a significant barrier to screening. What role can reimbursement programs play in offsetting these costs, making screening more accessible to women, especially those from lower socioeconomic backgrounds? | Thank you for insightful comment, The implication of the study findings have been added | Page No. 24 |
